# Yonsei criteria: a clinical reflection of stage I left-sided pancreatic cancer

**DOI:** 10.18632/oncotarget.22734

**Published:** 2017-11-28

**Authors:** Jae Uk Chong, Sung Hyun Kim, Ho Kyoung Hwang, Chang Moo Kang, Woo Jung Lee

**Affiliations:** ^1^ Division of Hepatobiliary and Pancreatic Surgery, Department of Surgery, Yonsei University College of Medicine, Seoul, Korea; ^2^ Pancreaticobiliary Cancer Clinic, Yonsei Cancer Center, Severance Hospital, Seoul, Korea

**Keywords:** pancreatic cancer, TNM staging, survival analysis, disease-free survival, pancreatectomy

## Abstract

In this study, we examined associations between pancreatic cancer that met the Yonsei criteria (YC) and classifications from the 8th edition of the American Joint Committee on Cancer (AJCC) staging system. Clinicopathological and survival data were collected from132 patients who underwent distal pancreatectomy for left-sided pancreatic ductal adenocarcinoma between January 2000 and December 2015, and the utility of the YC for selecting treatment and predicting survival was evaluated using the 8th AJCC staging manual. Of the 102 patients who ultimately qualified for the study, 45 patients were reclassified as stage I based on the 8th AJCC cancer staging system. Disease-free survival and disease-specific survival periods were longer in stage I patients who met the YC than in those who did not. Clinicopathological characteristics did not differ between stage I patients who did and did not meet the YC. These results suggest that meeting the YC criteria may be a clinical indicator that left-sided pancreatic cancer patients who are candidates for resection have early-stage disease according to the 8th edition of the AJCC staging manual.

## INTRODUCTION

Laparoscopic surgery has been accepted as the gold standard treatment in many oncologic fields [1–3]. Specifically, laparoscopic distal pancreatectomy is used for the treatment of benign and low-grade malignant pancreatic tumors. Several recent studies suggest that laparoscopic radical pancreatectomy could be also feasible and effective in treating pancreatic cancer [4–8]. However, the use of minimally-invasive radical pancreatectomy is still controversial because most patients are diagnosed with advanced-stage cancer that requires challenging surgical techniques; furthermore, this technique has not yet been evaluated in a randomized, controlled study.

Since 2007, our group has studied whether-minimally invasive pancreatectomy might be a safe and effective treatment for left-sided pancreatic cancer. Our results indicate that bloodless and margin-negative resection is crucial for treating pancreatic cancer arising from pancreatic body and tail lesions [9]. Based on this observation, we developed the following set of tumor conditions, termed Yonsei criteria (YC) [10, 11], to determine whether treatment with laparoscopic radical distal pancreatectomy is indicated: 1) tumor confined to the pancreas, 2) intact fascia layer between the distal pancreas and the left adrenal gland and kidney, and 3) tumor located more than 1–2 cm from the celiac axis. YC can be evaluated based on preoperative CT scans. For tumors that meet all three YC, currently available minimally-invasive techniques are highly safe and effective for achieving bloodless and margin-negative resection during the treatment of left-sided pancreatic cancer. In support of this hypothesis, our recent data demonstrate that long-term oncologic outcomes of minimally invasive radical pancreatectomy for left-sided pancreatic cancer that meets the YC were more favorable compared to those obtained with an open approach [12, 13]. Moreover, although surgical approach (minimally-invasive vs. open) is less predictive of oncologic outcome, YC strongly predicted long-term oncologic outcomes after radical pancreatectomy for left-sided pancreatic cancer. This suggests that YC is not only a potential indicator for minimally invasive radical pancreatectomy for treating left-sided pancreatic cancer, but may also represent some aspects of distal pancreatic cancer tumor biology [13].

Recently, the American Joint Committee on Cancer (AJCC) developed the 8th version of their cancer staging system for pancreatic cancer [14]. Unlike the 7th AJCC system [15], the new version states that T-stage is determined based on tumor size (T1: maximum tumor diameter ≤ 2 cm, T2: maximum tumor diameter >2 cm and ≤ 4 cm, T3: maximum tumor diameter >4 cm, and T4: unresectable primary tumor involving the celiac axis or the superior mesenteric artery), and N-stage has been recategorized based on the number of metastatic lymph nodes (N0: no regional lymph node metastasis, N1: metastasis in 1–3 regional lymph nodes, and N2: metastasis in ≥ 4 regional lymph nodes). According to the 7th AJCC cancer staging system, most left-sided pancreatic cancer that met the YC were categorized as T3-pancreatic cancer, and it was unclear why oncologic outcomes for pancreatic cancer that met the YC were superior to those that did not [12].

In this study, we examined associations between YC status and the 8th version of the AJCC cancer staging system categorizations to determine whether clinical YC classification is related to aspects of tumor biology in left-sided pancreatic cancer.

## RESULTS

### Oncologic outcomes for resected left-sided pancreatic cancer depending Yonsei criteria status

**Figure 1:**
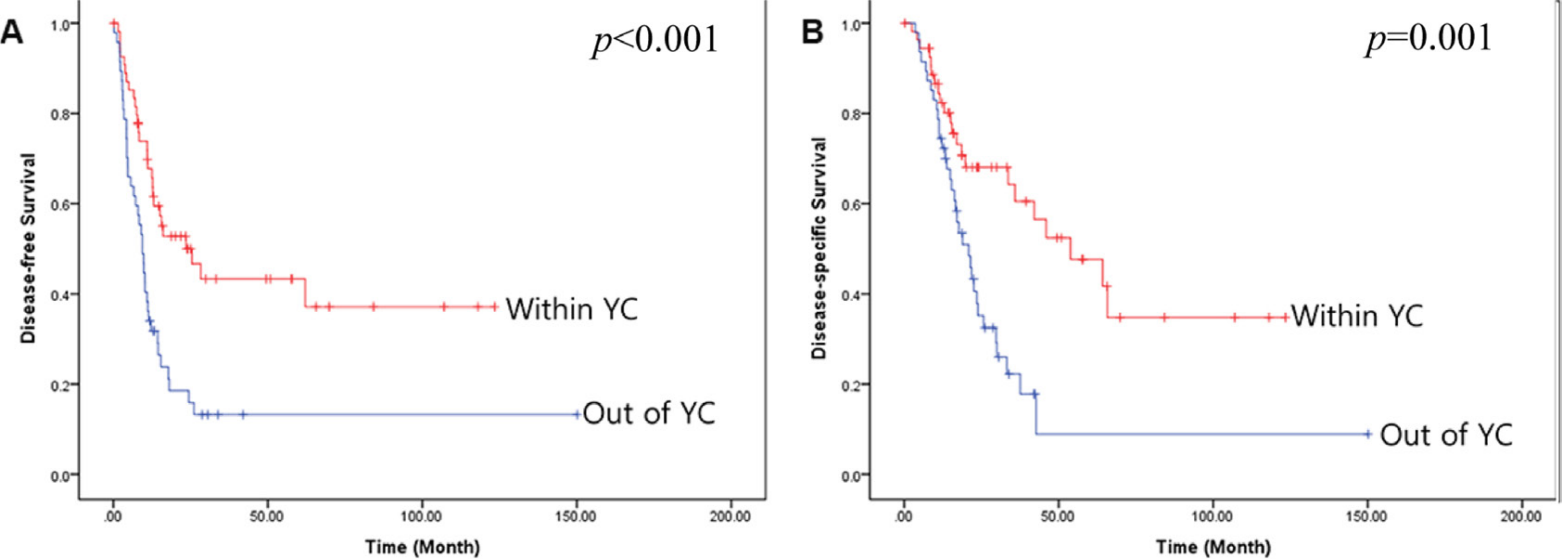
Oncologic outcomes in patients who do and do not meet the YC (**A**) Disease-free survival, (**B**) Disease-specific survival.

Among the 102 qualifying patients who underwent potentially curative resection of left-sided pancreatic cancer, 55 (53.9%) met the Yonsei criteria (YC) based on preoperative CT scans. Patients who did not meet the YC were much more likely to undergo combined organ resection (5/50 vs. 19/28, *p* < 0.001). Preoperative CT-based YC also strongly predicted long-term oncologic outcome. Mean disease-free (56.2 months [95% CI: 39.5–72.9] vs. 27.6 months [95% CI:12.8–42.3], *p* < 0.001, Figure 1A) and disease-specific survival (63.8 months [95% CI: 46.9–80.6] vs. 31.9 months [95% CI: 15.1–48.8], *p* = 0.001, Figure 1B) were longer in patients who met the YC than in those who did not.

### Associations between Yonsei criteria and AJCC cancer staging systems

According to 7th AJCC staging system, 94 patients (92.2%) were classified as T3. Additionally, most patients who met the YC were diagnosed with T3 pancreatic cancer (53 out of 57, 92.9%, Table 1). YC status was not correlated with T-stage from the 7th AJCC staging system (*p* = 0.425). However, YC was strongly associated with small-sized pancreatic cancer as described by the 8th AJCC cancer staging system (*p* = 0.019, chi-square, linear-to-linear association, Table 1). Tumor sizes were smaller in patients who met the YC, of whom 12 (21.1%) had pancreatic cancer tumors smaller than 2 cm, than in those who did not (2.9 ± 1.4 cm vs. 3.7 ± 1.7 cm, *p* = 0.012).

**Table 1:** T-stage distribution of resected-left sided pancreatic cancer

	7th AJCC staging system			*p*-value
	T1	T2	T3	
Within YC	0	4	53	
Out of YC	2	2	41	0.425

	8th AJCC staging system			*p*-value
	T1	T2	T3	
Within YC	12	39	8	
Out of YC	8	20	17	0.019

In contrast, YC was strongly associated with N-stage from 7th AJCC cancer staging system (*p* = 0.011). YC was also associated with small numbers of metastatic lymph nodes according to 8th AJCC cancer staging system (*p* = 0.015, chi-square, linear-to-linear association, Table 2); patients who met the YC had fewer lymph node metastases than those who did not (0.8 ± 1.5 vs. 2.0 ± 3.1, *p* = 0.037).

**Table 2:** N-stage distribution of resected-left sided pancreatic cancer

	7th AJCC staging system			*p*-value
	N0	N1		
Within YC	36	21		
Out of YC	17	28		0.011

	8th AJCC staging system			*p*-value
	N0	N1	N2	
Within YC	36	16	5	
Out of YC	17	20	8	0.015

### Prognosis prediction in resected left-sided pancreatic cancer using the 8th AJCC cancer staging system

**Figure 2:**
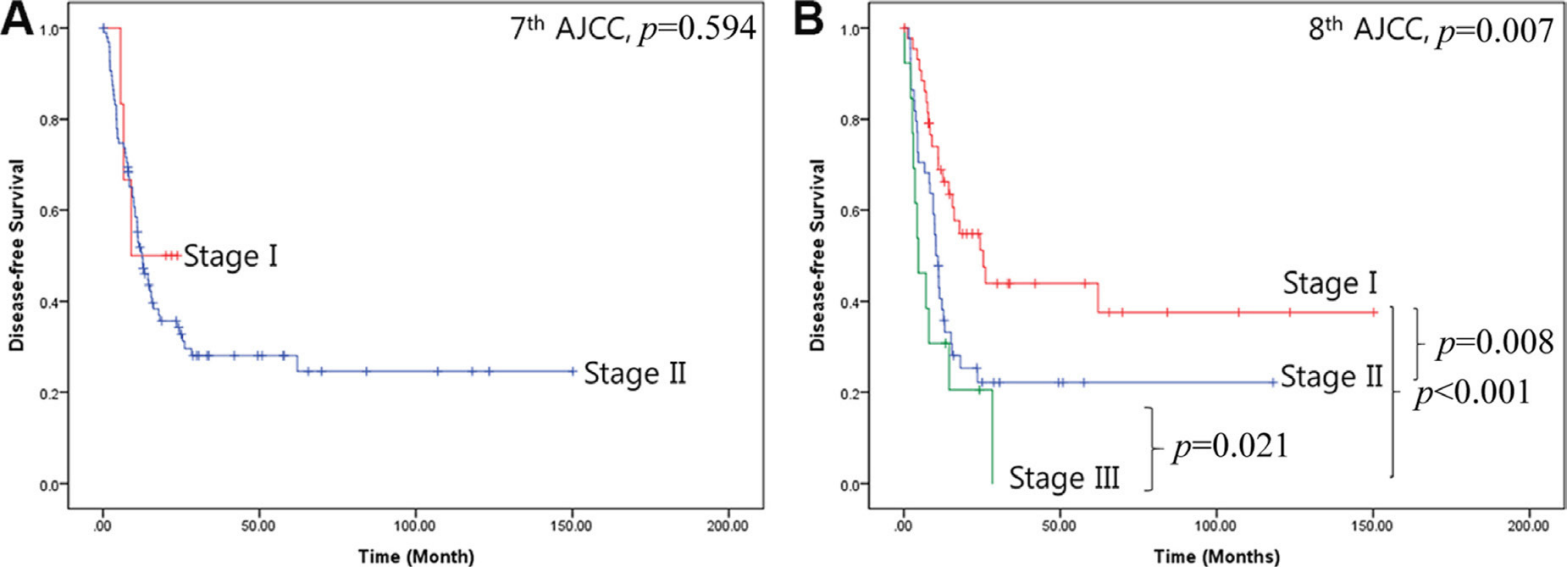
Disease-free survival for the two cancer staging systems (**A**) 7th AJCC cancer staging system, (**B**) 8th AJCC cancer staging system.

**Figure 3:**
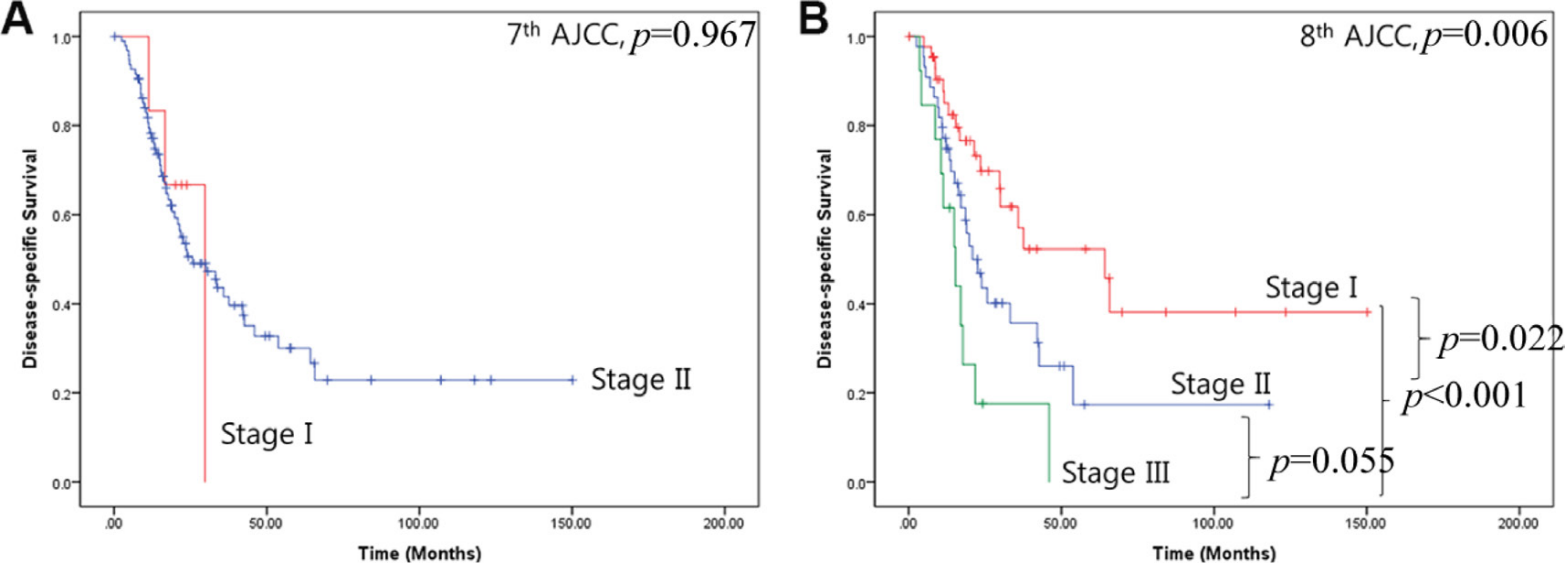
Disease-specific survival for the two cancer staging systems **(A)** 7th AJCC cancer staging system, **(B)** 8th AJCC cancer staging system.

As described above, most patients with resected left-sided pancreatic cancer were diagnosed with T3 disease according to 7th AJCC cancer staging system. It was therefore difficult to stratify the patient group because most had stage II cancer (Figure 2A and Figure 3A). However, prognostic differences became clear when the patients were classified according to 8th AJCC cancer staging system (Figure 2B, and Figure 3B); the 5-year disease-specific survival rate for stage I left-sided pancreatic cancer was 52.3%, with a mean survival time of 76 months [95% CI: 52.2–100.7].

### Stage-migration of patients meeting Yonsei criteria in the 8th AJCC cancer staging system

**Figure 4:**
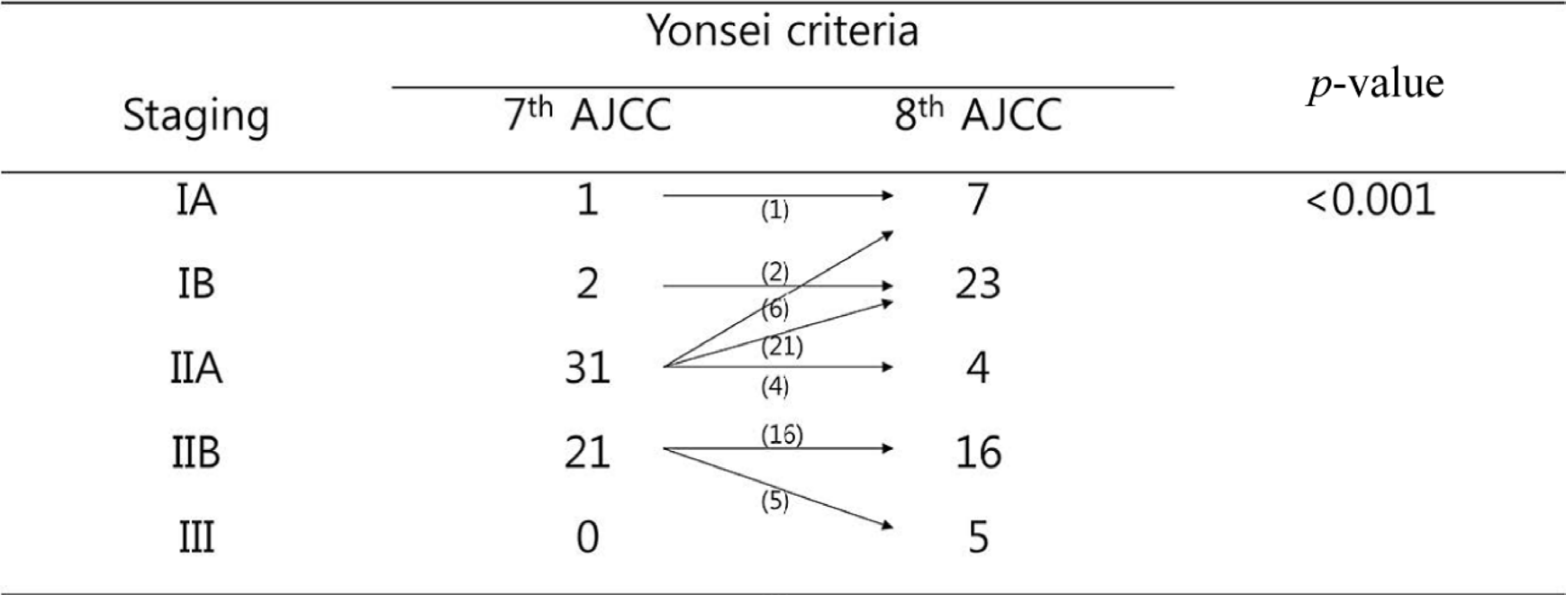
Stage-migration of left-sided pancreatic cancer that met the YC

Unlike the 7th AJCC cancer staging system, which classified most patients with resected left-sided pancreatic cancer who met the YC as stage II (52 out of 55 patients, 94.5%), 30 patients who met the YC (54.5%) were reclassified as stage I according to 8th AJCC cancer staging system; these patients comprised 66.7% of all patients with stage I resected left-sided pancreatic cancers (32 out of 45 patients). In addition, 87.1% of patients with stage IIA resected left-sided pancreatic cancer according to the 7th AJCC cancer staging system who met the YC (27 out of 31 patients) were reclassified as having stage I pancreatic cancer by the 8th AJCC cancer staging system (Figure 4).

### Yonsei criteria reflect clinically detectable early pancreatic cancer

**Figure 5:**
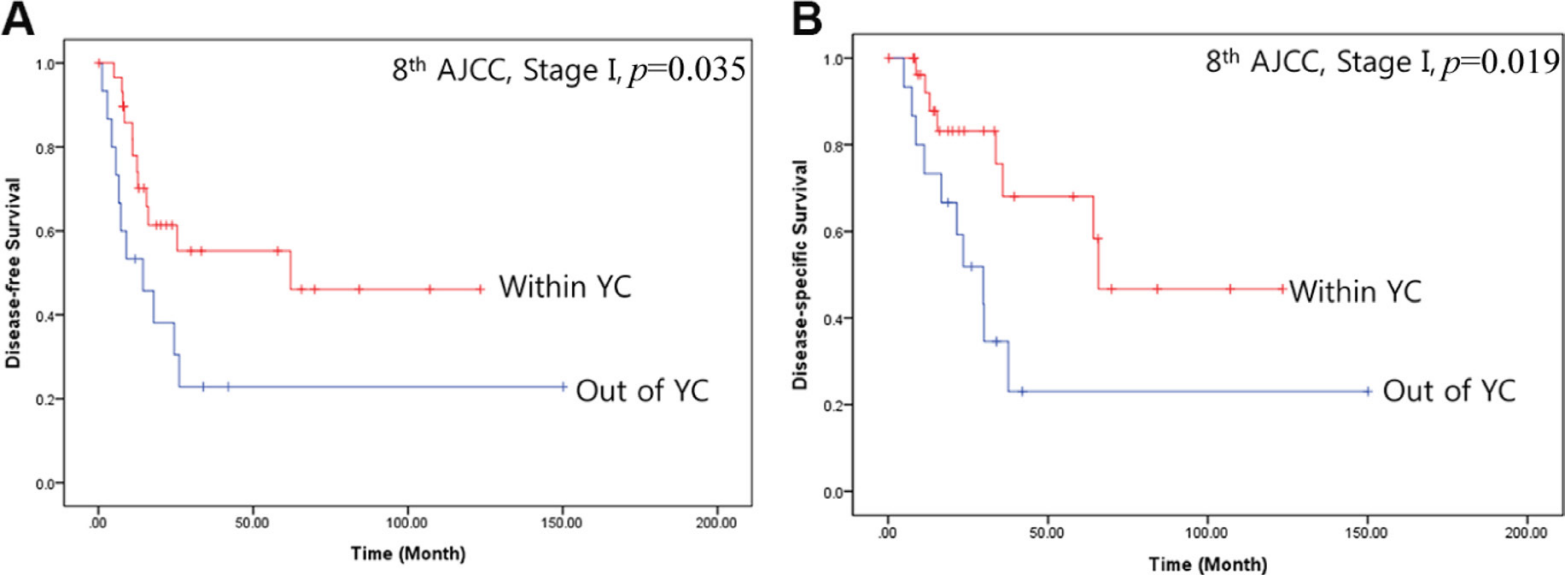
Oncologic outcomes for patients with stage I pancreatic cancer according to AJCC 8th staging system who do or do not meet the YC (**A**) Disease-free survival, (**B**) Disease-specific survival.

Of the 102 patients who underwent potentially curative resection for left-sided pancreatic cancer, 45 patients (44.1%) were reclassified as having stage I disease based on the 8th AJCC cancer staging system. Interestingly, oncologic outcome for stage I pancreatic cancer differed depending on YC status. Mean disease-free (68.4 months [95% CI: 45.6–91.10 vs. 43.1 months [95% CI: 12.0–74.1], *p* = 0.035, Figure 5A) and disease-specific survival (78.8 months [95% CI: 55.8–101.9] vs. 50.7 months [95% CI: 18.5–82.9], *p* = 0.019, Figure 5B) were longer in stage I pancreatic cancer patients who met the YC than in those who did not. Clinicopathological characteristics did not differ between stage I pancreatic cancer patients who did and did not meet the YC (Table 3).

**Table 3:** Clinico-pathological characteristics between stage I pancreatic cancer within Yonsei criteria and that out of Yonsei criteria

	8th AJCC-stage I pancreatic cancer		*p*-value
	Within YC (*N* = 30)	Out of YC ( *N* = 15)	
Gender (Male/Female)	18/12	8/7	0.670
Age, years	64.2 ± 7.5	64.3 ± 10.5	0.990
Symptoms (No/Yes)	15/15	3/12	0.105
Preoperative CA 19–9, U/mL	176.1 ± 306.6	167.2 ± 317.8	0.928
Tumor location	1/18/9/2	0/12/3/0	0.435
(Neck/Body/Tail/Body + Tail)			
Tumor size, cm	2.5 ± 0.8	2.9 ± 0.8	0.142
7th AJCC, pT-stage	1/2/27	0/3/12	0.330
(T1/T2/T3)			
Combined resection (No/Yes)	27/3	12/3	0.647
Differentiation	3/25/2	3/11/1	0.725
(WD/MD/PD)			
LVI	24/6	11/4	0.710
PNI	12/18	8/7	0.396
Retrieved LNs	15.0 ± 10.6	11.2 ± 9.1	0.220
R-status (R0/R1)	27/3	15/0	0.540
Postoperative complication (No/Yes)	21/9	6/9	0.105
POPF (No/Yes)	24/6	11/4	0.710
Postoperative adjuvant CTx (No/Yes)	7/22	5/10	0.516

## DISCUSSION

Based on our surgical experience with distal pancreatic cancer [9], we recently developed the YC as a potential indicator of the safety and efficacy of minimally-invasive pancreatectomy for left-sided pancreatic cancer [10, 11, 16]. We also demonstrated that, regardless of whether minimally-invasive surgery or open pancreatectomy was performed, left-sided pancreatic cancer patients who met the YC had more favorable long-term oncologic outcomes; propensity score matching analysis confirmed this result [12, 13]. The results of this study indicate that YC status may also reflect underlying tumor biology in pancreatic cancer patients when the 8th AJCC cancer staging system is used. YC status, which is based on preoperative CT scans, is thus clinical predictive factor of long-term survival outcomes for resected left-sided pancreatic cancer; most predictive factors in this disease are pathology-associated characteristics, such as lymph node metastasis [17], perineural invasion [18, 19], cell differentiation [20], and margin status [21], among others.

Here, we found that YC status was closely associated with T-stage in pancreatic cancers when the new tumor size guidelines were used (Table 1). Less extensive lymph node metastasis, as classified according to the 8th edition of the AJCC cancer staging manual, was also associated with cancers that met the YC (Table 2). Furthermore, 30 patients who met the YC (54.5%) were reclassified as having stage I pancreatic cancer (Figure 4), and 30 stage I pancreatic cancer patients (66.7%) met the YC, when the 8th AJCC cancer staging system was used. Although clinicopathological characteristics did not differ between stage I patients who did and did not meet the YC (Table 3), survival outcomes of stage I-distal pancreatic cancer patients differed depending on YC status (Figure 5). These findings suggest that YC itself may be a valuable clinical measure for predicting oncologic outcomes in patients with left-sided pancreatic cancer. As Rooij *et al.* [4] point out, evidence on the oncological feasibility of laparoscopic distal pancreatectomy is lacking; they therefore developed a decision-aid algorithm and proposed that small tumors and pT1/pT2 pancreatic cancer be treated by laparoscopic distal pancreatectomy. We previously developed YC to further assist in identifying patients who are eligible for a laparoscopic approach. According to the present results, most pancreatic cancer tumors that met the YC were relatively small and were classified as T1 or T2 pancreatic cancer using the 8th AJCC cancer staging system (Table 1). YC status may therefore be a reasonable indicator for the use of minimally-invasive radical pancreatectomy in left-sided pancreatic cancer and may be predictive of long-term survival. Additional studies should be performed in larger patient groups to confirm these results. Coincidentally, a recent ongoing randomized control trial has already identified YC as an inclusion criteria in diagnosing left-sided pancreatic cancer [22].

Several limitations of this study should be considered when interpreting the results. First, this study is retrospective. Second, the number of patients who met the YC is relatively small. Third, preoperative CT-based YC determinations may be subjective, and inter-observer biases might exist. Modifications to the YC might improve their ability to preoperatively define tumor characteristics more objectively, and thus more accurately represent tumor biology, in left-sided pancreatic cancer.

In summary, resected left-sided pancreatic cancer that meets the YC is a clinical marker of early (stage I) pancreatic cancer as classified by the 8th edition of AJCC cancer staging manual and is indicative of favorable tumor biology. In particular, stage I pancreatic cancer patients who meet the YC might be expected to have better long-term survival. This study provides a basis for additional investigations of left-sided pancreatic cancer patient subgroups that might benefit from minimally-invasive radical pancreatectomy.

## MATERIALS AND METHODS

Between January 2000 and December 2015, 132 patients received distal pancreatectomies to treat left-side pancreatic ductal adenocarcinoma. Among them, 27 patients who underwent resection following neoadjuvant chemotherapy with or without radiation and 3 patients who underwent palliative distal pancreatectomies were excluded. The medical records of the remaining 102 patients were retrospectively reviewed. Patients were categorized according to the YC [10, 11, 16], and the following clinicopathological data were collected: age, sex, presenting symptoms, serum CA 19–9, combined resection, adjuvant treatment, postoperative complication, cell differentiation, tumor size, T stage, N stage, number of retrieved lymph nodes (LN), number of positive LN, lymph node ratio, lymphovascular invasion, perineural invasion, and adjusted organ invasion. Survival data were obtained in institutional follow-ups for analysis of oncologic outcomes. The new 8th AJCC cancer staging manual [14] was used to classify patients and validate the YC. Disease-free survival and disease-specific overall survival periods were calculated from the date of operation to either the date recurrence was diagnosed or to the date of cancer-related death.

Statistical analyses were performed in IBM SPSS Statistics version 22. Continuous variables are represented as means ± standard deviations or medians with ranges, and categorical variables as percentages or frequencies. Student’s *t*-tests were applied for comparing continuous variables, and Fisher’s exact tests or Chi-squared tests were used for comparing categorical data. The Kaplan–Meier method was used for analysis of disease-free and overall survival. *P* < 0.05 was considered statistically significant. This study is a retrospective cohort study conducted at a single institution and was approved by the institutional review board.
